# The self-assembled nanoparticle-based multi-epitope influenza mRNA vaccine elicits protective immunity against H1N1 and B influenza viruses in mice

**DOI:** 10.3389/fimmu.2024.1483720

**Published:** 2024-10-08

**Authors:** Yaxin Di, Chenchao Zhang, Zilin Ren, Renyue Jiang, Jiafeng Tang, Songhui Yang, Ziliang Wang, Tong Yu, Tong Zhang, Ziping Yu, Zhiqiang Xu, Xinyu Zhuang, Ningyi Jin, Mingyao Tian

**Affiliations:** ^1^ College of Veterinary Medicine, Northeast Agricultural University, Harbin, China; ^2^ Changchun Veterinary Research Institute, Chinese Academy of Agricultural Sciences, State Key Laboratory of Pathogen and Biosecurity, Key Laboratory of Jilin Province for Zoonosis Prevention and Control, Changchun, China; ^3^ College of Agriculture, Yanbian University, Yanji, China; ^4^ College of Animal Science and Technology, Guangxi University, Nanning, China; ^5^ College of Veterinary Medicine, Jilin Agricultural University, Changchun, China; ^6^ College of Veterinary Medicine, Jilin University, Changchun, China; ^7^ Jiangsu Co-innovation Center for Prevention and Control of Important Animal Infectious Diseases and Zoonoses, Yangzhou University, Yangzhou, China

**Keywords:** influenza virus, multivalent epitopes, nanoparticle, mRNA vaccines, immunogenicity

## Abstract

**Introduction:**

The influenza virus is recognized as the primary cause of human respiratory diseases, with the current influenza vaccine primarily offering strain-specific immunity and limited protection against drifting strains. Considering this, the development of a broad-spectrum influenza vaccine capable of inducing effective immunity is considered the future direction in combating influenza.

**Methods:**

The present study proposes a novel mRNA-based multi-epitope influenza vaccine, which combines three conserved antigens derived from the influenza A virus. The antigens consist of M2 ion channel’s extracellular domain (M2e), the conserved epitope of located in HA2 of hemagglutinin (H1, H3, B), and HA1 of hemagglutinin. At the same time, trimeric sequences and ferritin were conjugated separately to investigate the immune effects of antigen multivalent presentation.

**Results:**

Immunization studies conducted on C57BL/6 mice with these vaccines revealed that they can elicit both humoral immunity and CD4^+^ and CD8^+^ T cell responses, which collectively contribute to enhancing cross-protective effects. The virus challenge results showed that vaccinated groups had significantly reduced lung damage, lower viral loads in the lungs, nasal turbinates, and trachea, as well as decreased levels of pro-inflammatory cytokines.

**Conclusion:**

These findings clearly demonstrate the wide range of protective effects provided by these vaccines against H1N1 and B influenza viruses. The present finding highlights the potential of mRNA-based influenza vaccines encoding conserved proteins as a promising strategy for eliciting broad-spectrum protective humoral and cellular immunity against H1N1 and B influenza viruses.

## 1 Introduction

Influenza is an infectious viral disease caused by the influenza virus, which poses a significant global health threat due to its high morbidity and mortality rates (1). The World Health Organization (WHO) reports an annual incidence of 3 to 5 million cases for this disease, resulting in an approximate mortality rate of 10% (2). Vaccination as a key and proven method for preventing influenza infections among the population (3). However, the continuous antigenic evolution and transmission of influenza viruses often lead to a discrepancy between the annual influenza vaccines recommended by the WHO and the circulating viral strains, thereby compromising the efficacy of these vaccines in combating infection (4–6). Therefore, it is crucial to develop a universal influenza vaccine that can provide cross-protection against various strains of the virus.

The development of universal influenza vaccines is currently being consideration, with various strategies being explored. One promising approach involves using a novel epitope-based platform for vaccination against influenza viruses (7). Utilizing a single short epitope may lead to limited vaccine efficacy, whereas an alternative approach entails the amalgamation of multiple conserved epitopes within a singular vaccine (8). This approach aims to enhance protective immunity and prevent viruses from evading vaccine-induced immunity. A study by Sang-Moo Kang et al. demonstrated that a vaccine incorporating the HA2 and M2e genes effectively protected adult and aged mice against different subtypes of heterologous and heterosubtypic cross-group viruses at similar levels (9, 10).

Epitope-based vaccines have been reported to elicit a weaker immune response compared to conventional vaccines (11). Therefore, current research is focused on developing strategies to enhance the immunogenicity of epitope vaccines. One approach involves incorporating heterotrimeric motifs into HA’s structure to mimic its natural conformation and improve stability (12). Another promising method is using nanoplatforms for delivering relevant antigens, which shows potential in the development of new influenza vaccines (13). For example, Zykova AA et al. developed a recombinant protein containing tandem copies of M2e and HA2 fused with artificial self-assembled peptides, demonstrating its ability to induce strong humoral and cellular immunity in mice (14). Similarly, Qiao et al. combined the A helix (Ah) and CD helix (CDh) from the H3N2 virus HA stem with ferritin for immunization studies on mice, finding that formulations of CDh-f and (A+CD) hf induced robust humoral and cellular immune responses, providing protection against lethal infections caused by the H3N2 virus (15). Additionally, Pan et al. designed MHNF nanoparticles comprising the A α-helix of hemagglutinin (H), the ectodomain of matrix protein 2 (M), and the HCA-2 of neuraminidase (N), which were conjugated with self-assembling recombinant human heavy chain ferritin cages. This vaccine was found to stimulate high levels of antigen-specific antibodies and cellular immune responses, offering protection against various influenza viruses (16).

Nucleoside modified mRNA-LNP vaccines have emerged as an attractive platform for controlling infectious diseases, especially during the COVID-19 pandemic. These vaccines introduce messenger RNA (mRNA) encoding antigenic proteins into the host organism, leading to the production of corresponding antigens and triggering specific immune responses (17). Significantly, mRNA vaccines have played a crucial role in effectively addressing the COVID-19 crisis. The Pfizer-BioNTech (BNT162b2), Moderna (mRNA-1273), and CureVac vaccines, developed using mRNA technology, represent an unprecedented milestone in medical vaccine development (18–20). The mRNA vaccines have not only been effective against COVID-19 but also gained attention for their potential in other viral research areas. For example, Zhuang et al. successfully developed an mRNA vaccine against the H1N1 virus and tested it on mice *in vivo*. The study showed that the mRNA vaccine effectively protected the mice from influenza virus infection by inducing strong immune responses (21). Additionally, Freyn et al. engineered a modified mRNA-LNP vaccine incorporating HA stem, NA, M2e, and NP antigen proteins. Subcutaneous immunization of mice with this vaccine elicited a robust and broad immune response. Post-immunization serum antibodies demonstrated efficacy against diverse influenza virus strains, highlighting the potential of this nucleoside-modified mRNA-LNP vaccine expressing multiple conserved antigens as a promising candidate for a universal influenza virus vaccine (22).

The mRNA platform presents an appealing approach for vaccine development, offering distinct advantages over conventional platforms in terms of safety, expedited development process, robust immunogenicity, streamlined vaccine design, and simplified manufacturing. In this study, we present the efficacy and immunogenicity evaluation of a universal influenza mRNA vaccine targeting conserved epitopes. Our findings demonstrate a potent antigen-specific humoral and cellular immune response induced by the vaccine, conferring protection against both H1N1 and B subtypes of influenza viruses in mice models.

## 2 Materials and methods

### 2.1 Mice, cells, and viruses

Female C57BL/6 mice, aged 6 weeks, were procured from BEIJING HFK BIOSCIENCE Co., Ltd. The HEK-293T cells and PGEM-3Zf-n3 vector were maintained in our laboratory. HEK-293T cells were cultured in Dulbecco’s modified Eagle’s medium (DMEM) supplemented with 10% fetal bovine serum (FBS, Gibco) and 100 units/mL penicillin-streptomycin. Our laboratory possessed the following virus strains: A/Puerto/R/8/34(H1N1), A/Jilin/JYT-01/2018(H1N1), B/Massachusetts/2/2012(Yamagata), and B/Jilin/02/2022(Victoria). All experiments involving live viruses were conducted in biosafety level 2 facilities.

### 2.2 mRNA preparation *in vitro*

**Figure 1:**
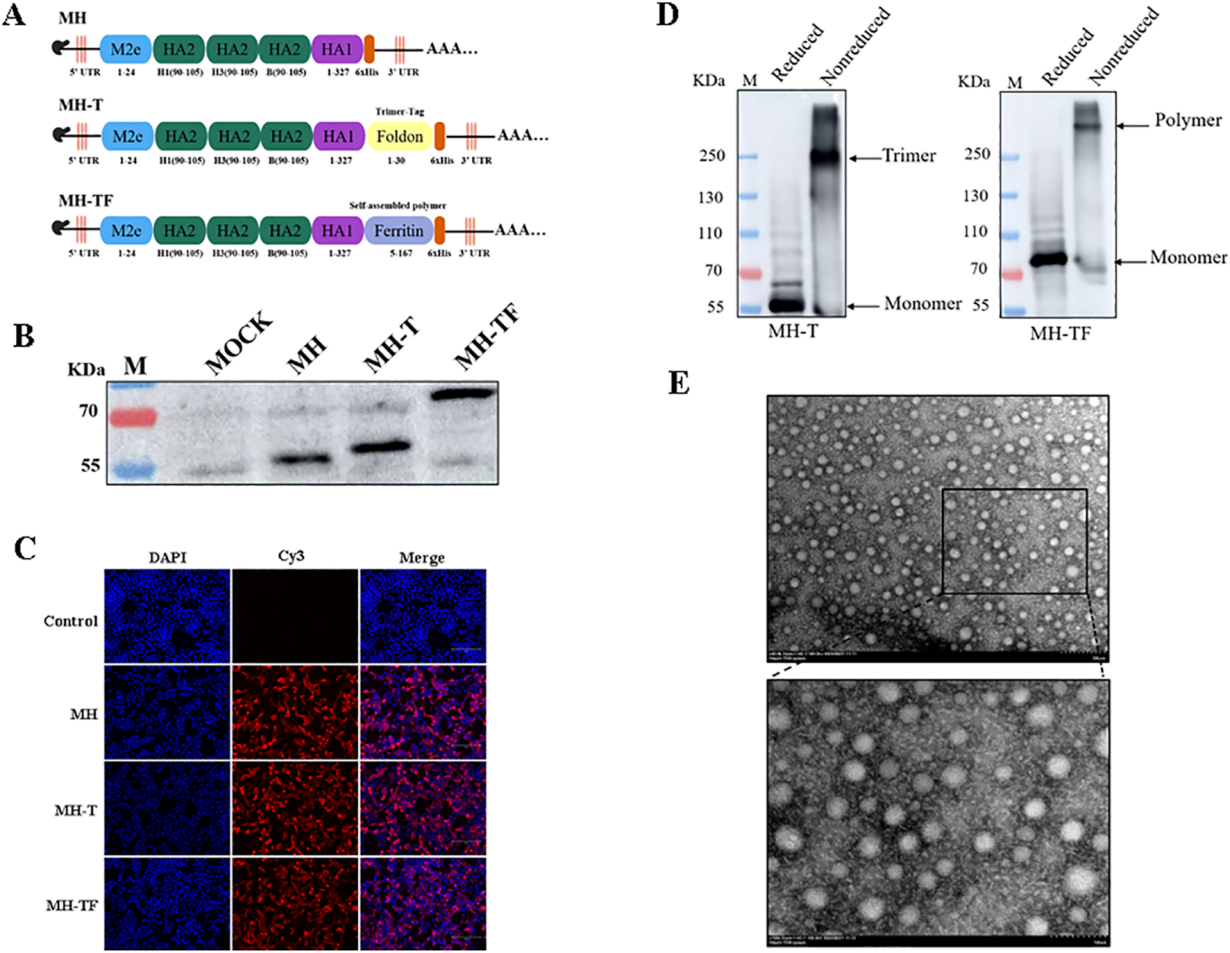
Construction and expression of the candidate mRNA vaccines. **(A)** Structural diagram of mRNA candidate vaccines. **(B)** Verification of intracellular protein expression in transfected cells which treated with reducing loading buffer. **(C)** Verification of the expression of protein by Indirect immunofluorescence. **(D)** Verification of intracellular protein expression treated with SDS-PAGE loading buffer (non-reduced). **(E)** TEM images of MH-TF protein, Scale: 200 nm/100 nm.

The HA2 domain, which contains epitopes targeted by broadly neutralizing antibodies, holds promise as a potential candidate for the development of vaccines with broad protective efficacy. In this study, we focused on screening HA2 epitopes that are validated and relatively conserved B-cell dominant epitopes (23). Additionally, M2e also is considered an important target for designing influenza vaccines due to its highly conserved nature across multiple strains of the virus. To obtain mRNA vaccine for subsequent *in vivo* utilization, the peptide sequences of M2e, HA2, and other relevant sequences were documented in **Supplementary Table 1** and **Figure 1A**, along with their respective gene order. The aforementioned genes were subsequently cloned into the pGEM-3Zf-n3 vector utilizing *Cla* I and *Pac* I restriction enzymes (24). The resulting plasmids were subsequently linearized by *Xho* I cleavage in preparation for their use as DNA templates in an *in vitro* transcription reaction. The T7-FlashScribe Transcription Kit (CELLSCRIPT, USA) was used to generate mRNA from these DNA templates. To enhance *in vivo* stability, resistance to degradation, and translational efficiency, 1-Methylpseudouridine-5′-triphosphate (TriLink, USA) was employed as a substitute for uridine triphosphate (UTP). Subsequently, the mRNA molecules were purified using the MEGAclear Transcription Clean-Up Kit (Thermo Fisher Scientific, USA) and capped utilizing the ScriptCap Cap 1 Capping System (CELLSCRIPT, USA).

### 2.3 mRNA expression

After seeding HEK293T cells in 12-well plates and allowing them to grow overnight until they reached approximately 80-90% confluence, the cells were transfected with mRNA using the Xfect RNA Transfection Reagent protocol (TAKARA, Japan). Cell lysates were collected at 24 hours post-transfection using RIPA lysis buffer for protein expression confirmation through western blotting with Influenza A Virus Hemagglutinin/HA Antibody, Rabbit Mab (Sino Biological, China), while mock-transfected cells served as controls. Indirect immunofluorescence technology was used to assess corresponding protein expression levels. The structure of MH-TF protein was observed by HT7800 transmission electron microscope.

### 2.4 Vaccination and virus challenge

A total of 36 female C57BL/6 mice, aged 6 weeks, were randomly divided into three groups for vaccine immunization and a negative control group, with each group consisting of 9 mice. Intramuscular injections were administered every two weeks for a total of three doses. Serum samples were collected at 7, 14, 21, 28, 35 and 42 days after the primary immunization and stored at −20°C for further analysis. On the 42^nd^ day following the primary immunization, mice were challenged with influenza virus (10×LD_50_). Subsequently, body weight and survival of the mice were monitored daily for two weeks post-virus challenge.

### 2.5 Enzyme-linked immunosorbent assay

The plates were coated with recombinant protein (5 μg/mL, 100 μL/well) at 4°C, followed by blocking with 3% bovine serum albumin for 2 hours. Subsequently, sera samples (a dilution of 1:100) were added and incubated at 37°C for 1.5 hours. Afterwards, the plates were washed and incubated with Goat Anti-Mouse IgG Human ads-HRP (SouthernBiotech, USA). The signal was developed using TMB as the substrate, and the reaction was terminated by adding 2M H_2_SO_4_. Finally, the optical density at 450 nm (OD_450_) was measured using a microtiter plate reader.

### 2.6 Hemagglutination inhibition assays

Hemagglutination inhibition (HI) assay was measured as described earlier (25, 26). The immune serum samples were pretreated with receptor destroying enzyme II and diluted twofold before detecting HI in 96-well plates. Each well was incubated with 4 HA units of virus at room temperature for 30 min, followed by mixing with 1% chicken red blood cells and further incubation for another 30 min. The HI titer is defined as the reciprocal of the maximum serum dilution that inhibits erythrocyte viral hemagglutination.

### 2.7 Flow cytometry

The spleens of mice were harvested on the 42^nd^ day after the first vaccination using aseptic techniques. Single-cell suspensions were prepared by passing them through a 70 μm cell strainer and treating them with Red Blood Cell Lysis Buffer (Beyotime, China). The cells were then stimulated with the recombinant protein and treated with Brefeldin A Solution (1X) for 5 h. After stimulation, the cells were washed with PBS buffer and stained with FITC-conjugated anti-mouse CD3, APC-conjugated anti-mouse CD4, and PE-conjugated anti-mouse CD8a antibodies (BioLegend, USA). Following staining, the cells were fixed using Cytofix/Cytoperm™ Solution and labeled with PE-conjugated anti-mouse IFN-γ antibodies (BioLegend, USA). Finally, the stained cells were washed twice and analyzed using a flow cytometer (Beckman Coulter, USA) to detect immune responses mediated by antigen-specific CD4^+^ and CD8^+^ T lymphocytes.

### 2.8 Viral load and cytokines

On the 5^th^ day post-virus challenge, we collected nasal turbinates, trachea, and right lung from each experimental group to measure viral load. Total cellular RNA was extracted from homogenized supernatants using the QlAamp Viral RNA Mini Kit (Qiagen, Germany) according to the manufacturer’s instructions. The viral load and expression of cytokines (IL-1β, IL-6, TNF-α) were quantified using the HiScript//one-step qRT-PCR SYBR Green kit (Vazyme, China). Primer sequences used in this assay can be found in **Supplementary Table 2.**

### 2.9 Histopathology

On the 5^th^ day post-virus challenge, the mice were euthanized and their left lung lobes were fixed in 4% paraformaldehyde, embedded in paraffin, and subjected to H&E staining for histopathological examination to evaluate lung injury.

### 2.10 Statistical analysis

The data were presented as mean ± standard error of the mean (SEM). To determine statistically significant differences in group means, a one-way analysis of variance (ANOVA) with Tukey’s multiple comparison tests was performed using GraphPad Prism software. In the figures, significance levels were denoted as **** *p* < 0.0001, *** *p* < 0.001, ** *p* < 0.01, * *p* < 0.05, ns *p* > 0.05.

## 3 Results

### 3.1 Construction and expression of influenza mRNA vaccines

The immunogenicity of epitopes often results in comparatively weaker immune responses when compared to conventional vaccines, thereby limiting the efficacy of epitope-based vaccines. In this study, we developed three mRNA vaccines targeting influenza viruses: a monomeric structure (MH), a trimeric structure (MH-T), and an architecture based on ferritin (MH-TF) (**Figure 1A**). The successful expression of proteins encoded by the mRNA for MH, MH-T, and MH-TF immunogens was confirmed through Western blot analysis treated with reducing loading buffer (**Figure 1B**) and indirect immunofluorescence (**Figure 1C**) performed on the transfected cell lysates. Concurrently, non-reductive treatment was employed to examine the expression patterns of MH-T and MH-TF proteins (**Figure 1D**), TEM images analysis of MH-TF revealed nanoparticles with a diameter of nearly 50 nm (**Figure 1E**).

### 3.2 Humoral immune responses of mRNA vaccines

**Figure 2:**
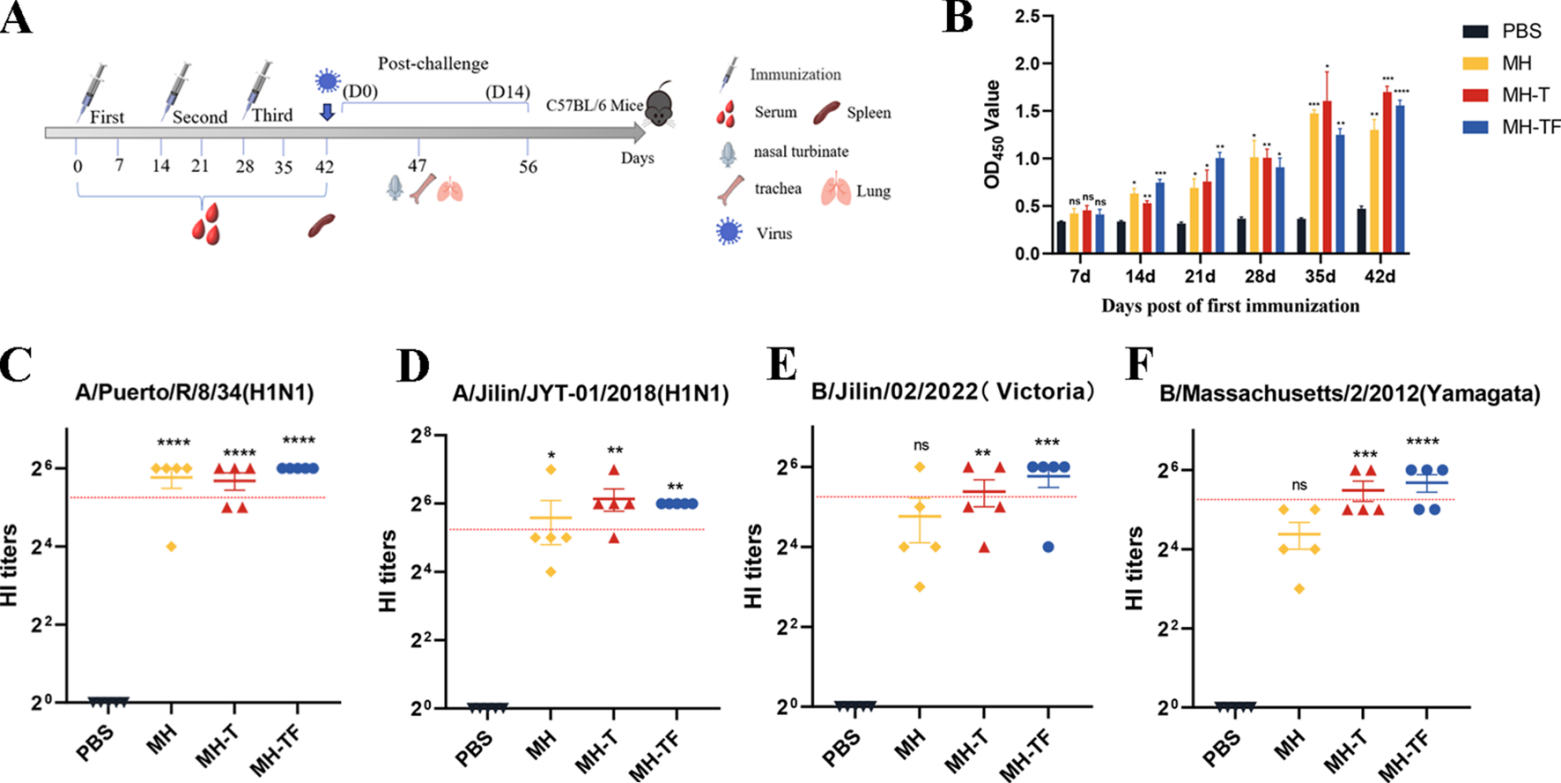
Antibody level analysis of vaccinated mice. **(A)** Schematic diagram of immunization of C57BL/6 mice. The number of immunizations was 3 doses, and the immunization interval was 14 days. **(B)** ELISA detection of IgG levels specific in the serum of C57BL/6 mice at various time points post-immunization. **(C)** HI antibody titers (A/Puerto/R/8/34(H1N1)) in mice immunized with different vaccines on 42 days. **(D)** HI antibody titers (A/Jilin/JYT-01/2018(H1N1)) in mice immunized with different vaccines on 42 days. **(E)** HI antibody titers (B/Jilin/02/2022(Victoria)) in mice immunized with different vaccines on 42 days. **(F)** HI antibody titers (B/Massachusetts/2/2012(Yamagata)) in mice immunized with different vaccines on 42 days. The red dotted line represents an antibody titer value of 40. Data are mean ± SEM, analyzed using one-way ANOVA (****p < 0.0001, ***p < 0.001, **p < 0.01, *p < 0.05, ns *p* > 0.05).

The humoral immune response is crucial for preventing virus entry and enhancing virus elimination. This study assessed the antibody levels produced by influenza virus mRNA vaccines in C57BL/6 mice. The humoral immune response of the mRNA vaccine was evaluated by administering three doses, each at a dose of 15 μg, to female C57BL/6 mice (n = 9), with a two-week interval between each vaccination(**Figure 2A**). Enzyme-linked immunosorbent assay (ELISA) was employed to assess specific levels of immunoglobulin G (IgG) in serum samples collected on days 7, 14, 21, 28, 35, and 42 after the initial vaccination. All three vaccines induce specific antibodies within 14 days of the initial vaccination, with antibody titers peaking on day 42 in particular(**Figure 2B**). The OD_450_ values of MH, MH-T and MH-TF were significantly higher than those of the PBS group (*p*=0.007, *p*=0.0005, and *p*<0.0001). The evaluation of hemagglutination inhibition activity facilitates the identification of antibodies that specifically bind to the receptor binding domain of hemagglutinin. Generally, an HI titer equal to or greater than 40 is considered indicative of a protective effect. 42 days after the first immunization, compared to the antibody sera from the PBS group, significant HI activity was observed in the antibody sera from MH, MH-T, and MH-TF groups, with the highest HI titer detected in the MH-TF group (**Figures 2C–F**). However, no significant difference in HI titers targeting influenza B viruses was found between the MH group and PBS group (**Figures 2E–F**).

### 3.3 Cellular immune responses of mRNA vaccines

**Figure 3:**
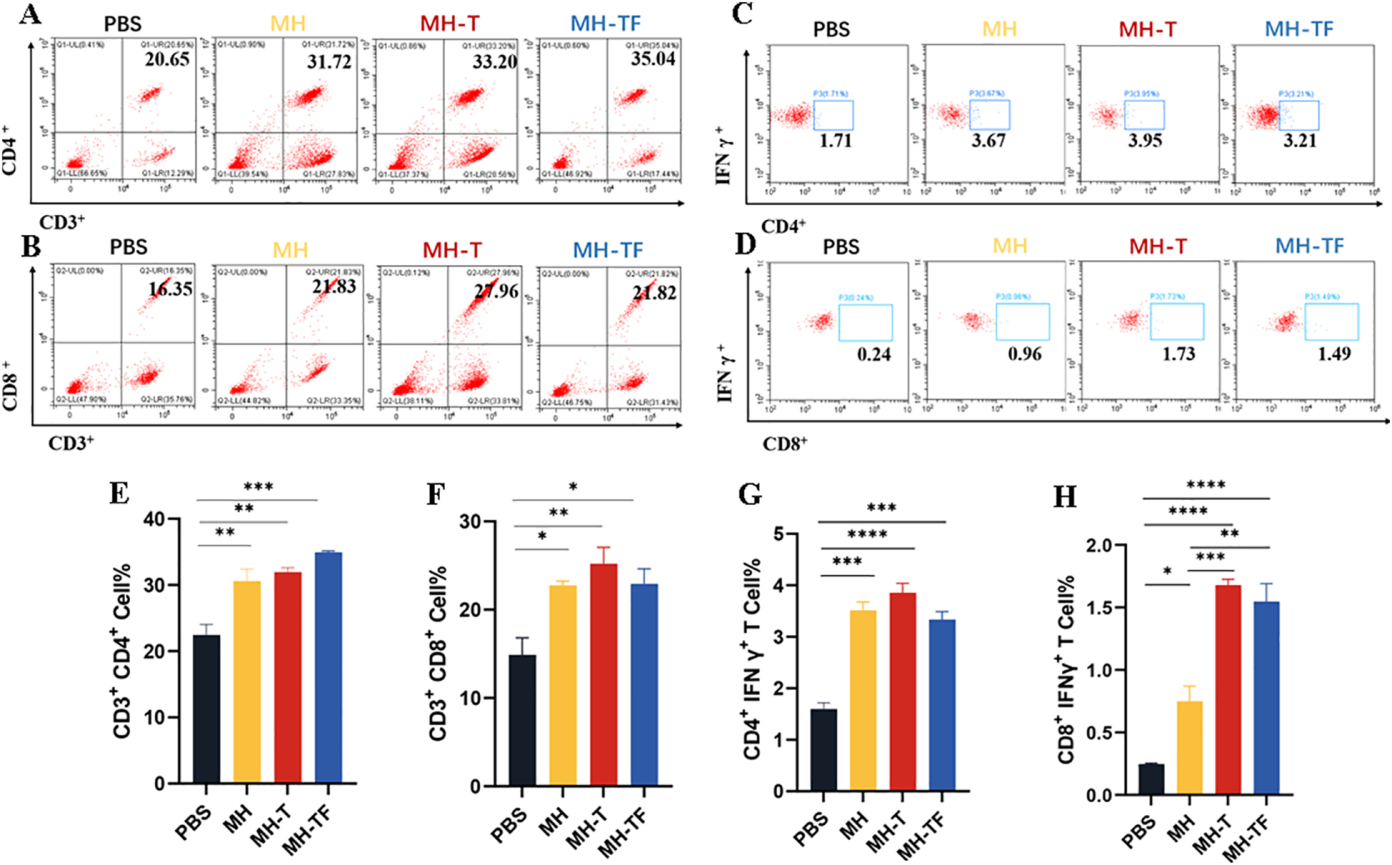
Cellular immune analysis of vaccinated mice. **(A, B)** The typical flow cytometry analysis of CD3^+^, CD4^+^ and CD3^+^, CD8^+^ T cells. **(C, D)** The typical flow cytometry analysis of IFN-γ^+^ in CD4^+^ and CD8^+^ T cells. **(E, F)** Percentage of CD3^+^, CD4^+^ and CD3^+^, CD8^+^ T cells within splenic lymphocyte population. **(G, H)** Percentage of IFN-γ^+^ in CD4^+^ and CD8^+^ T cell responses. Data are mean ± SEM, analyzed using one-way ANOVA (****p < 0.0001, ***p < 0.001, **p < 0.01, *p < 0.05).

We further elucidated the cellular immune response elicited by mRNA-based vaccines. T lymphocytes isolated from the spleen of immunized and control mice (n=3) two weeks after the third immunization were assayed using ICS assay. All the three vaccine groups elicited antigen-specific CD4^+^ (**Figure 3A, E**) and CD8^+^ (**Figure 3B, F**) T cells. At the same time, the level of IFN-γ produced by CD3^+^ CD4^+^ T cells (**Figure 3C, G**) and CD3^+^ CD8^+^ T cells (**Figure 3D, H**) in three vaccine groups were found to be significantly higher compared to those in the control group. Notably, The level of IFN-γ produced by CD3^+^ CD8^+^ T cells in MH-T and MH-TF groups was significantly increased compared with that in MH group, and was 2.24 times and 2.06 times, respectively. These findings suggest that vaccination elicits augmented cellular immune response.

### 3.4 Protection of mRNA vaccines from influenza virus challenge in mice

**Figure 4:**
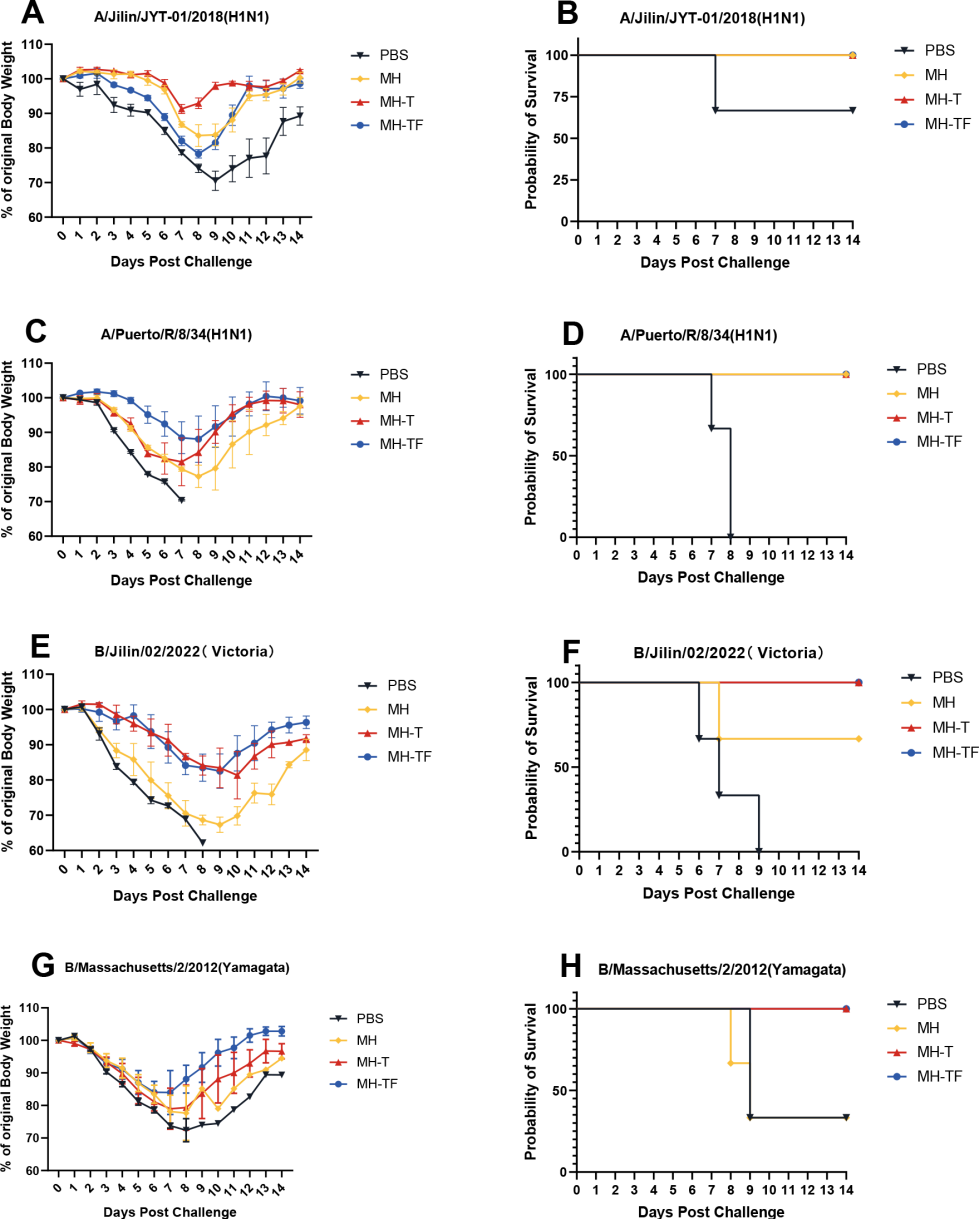
Body weight changes and survival curves of influenza virus-infected mice. Mice were challenged with 10 x LD_50_ of A/Jilin/JYT-01/2018(H1N1) **(A, B)** and A/Puerto/R/8/34(H1N1) **(C, D)** and B/Jilin/02/2022(Victoria) **(E, F)** and B/Massachusetts/2/2012(Yamagata) **(G, H)**. Body weight (left) and survival rate (right) were monitored daily during 14 days.

**Figure 5:**
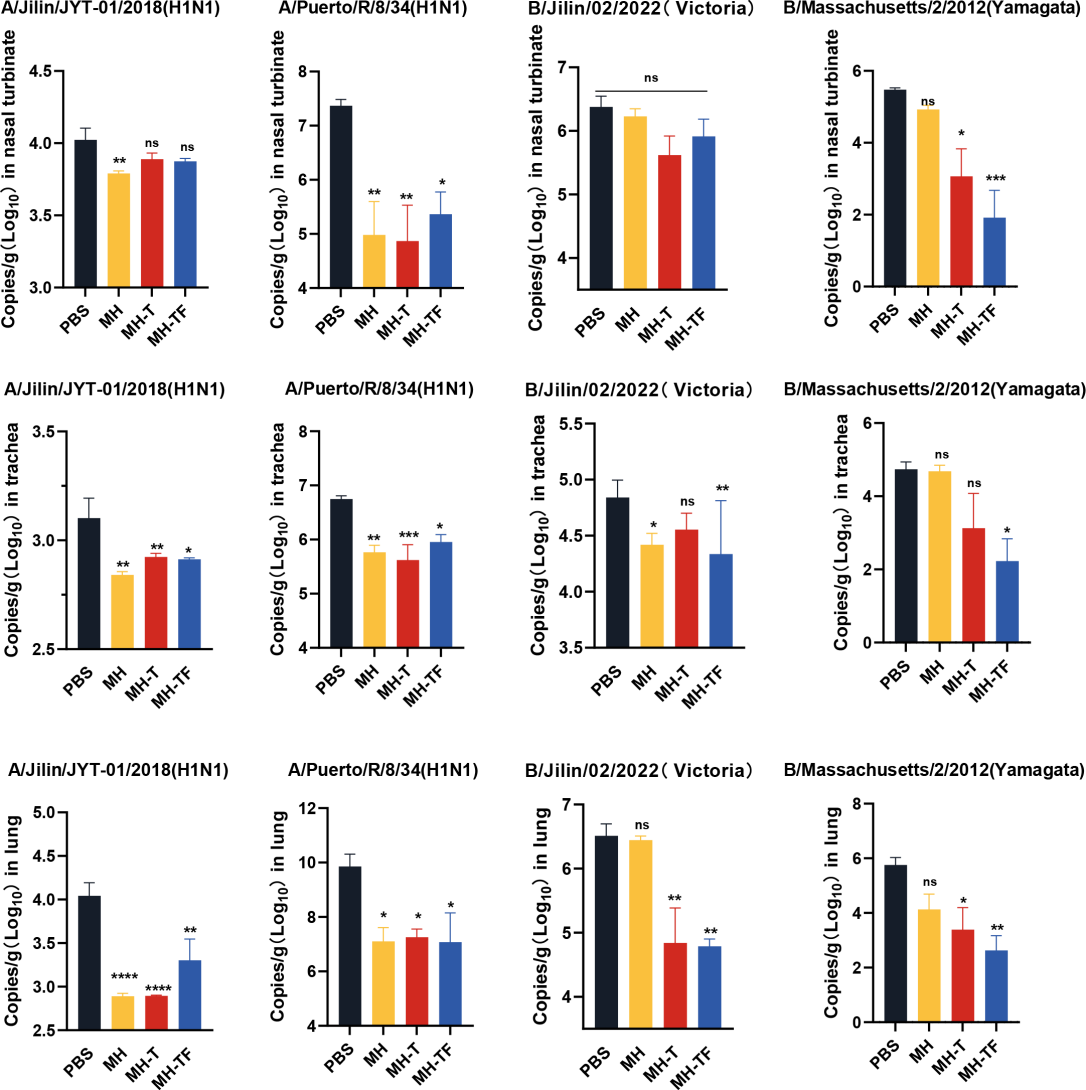
Analysis of viral loads after influenza virus challenge. Quantification of viral loads for the HA gene in nasal turbinate, trachea and lung. Data are mean ± SEM, analyzed using one-way ANOVA (****p < 0.0001, ***p < 0.001, **p < 0.01, *p < 0.05, ns *p* > 0.05).

42 days after the first immunization, the mice were challenged intranasally with 10× LD_50_ influenza virus. The weight and survival rate of the mice were monitored daily for two weeks(**Figure 4**). The results showed that mRNA vaccine-immunized mice exhibited protective effects against weight reduction and death, whereas control mice succumbed to the virus attack, and the protective efficiency of MH-T and MH-TF groups was 100%. On day 5 post-infection, viral load of the turbinate, trachea, and lungs exhibited a significantly diminished in the vaccinated group compared to the control group (**Figure 5**).

### 3.5 Pathological section analysis

**Figure 6:**
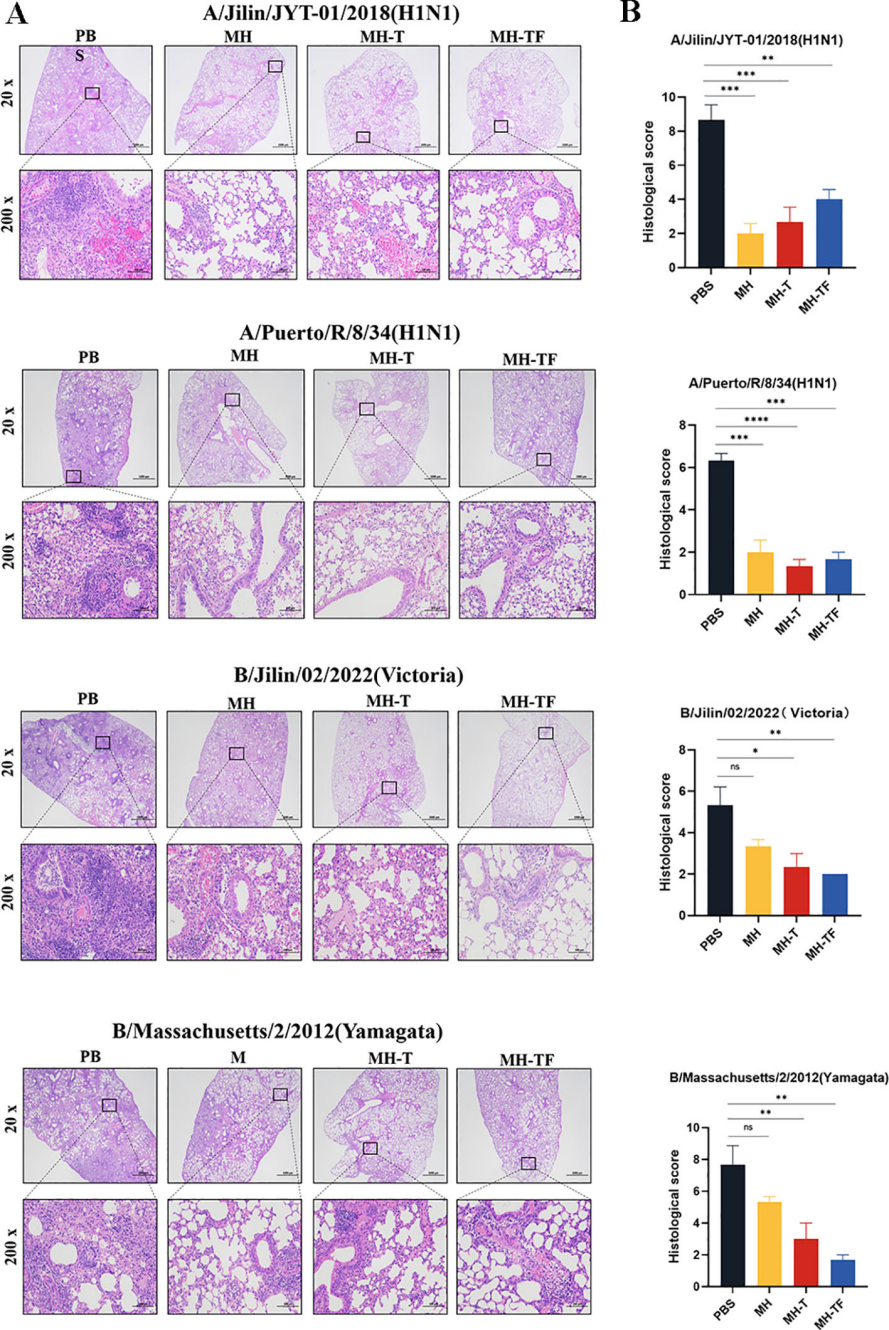
Lung physiology of influenza virus-infected mice. **(A)** Representative images of H&E-stained lung sections on the 5^th^ day postinfection (dpi), n=3 for each group. **(B)** Results of histology scores. Data are mean ± SEM, analyzed using one-way ANOVA (****p < 0.0001, ***p < 0.001, **p < 0.01, *p < 0.05, ns *p* > 0.05).

The histological examination of lung tissue revealed a significant reduction in lung injury among vaccinated mice, accompanied by decreased inflammatory cell infiltration, preserved alveolar structure integrity, slightly thickened alveolar walls, and reduced epithelial cell necrosis (**Figure 6A**). To visually compare the variations in pathological damage across each group, we evaluated the outcomes of the pathological sections. A higher score indicates a more pronounced level of pathological damage (**Figure 6B**).

### 3.6 Cytokine gene expression

**Figure 7:**
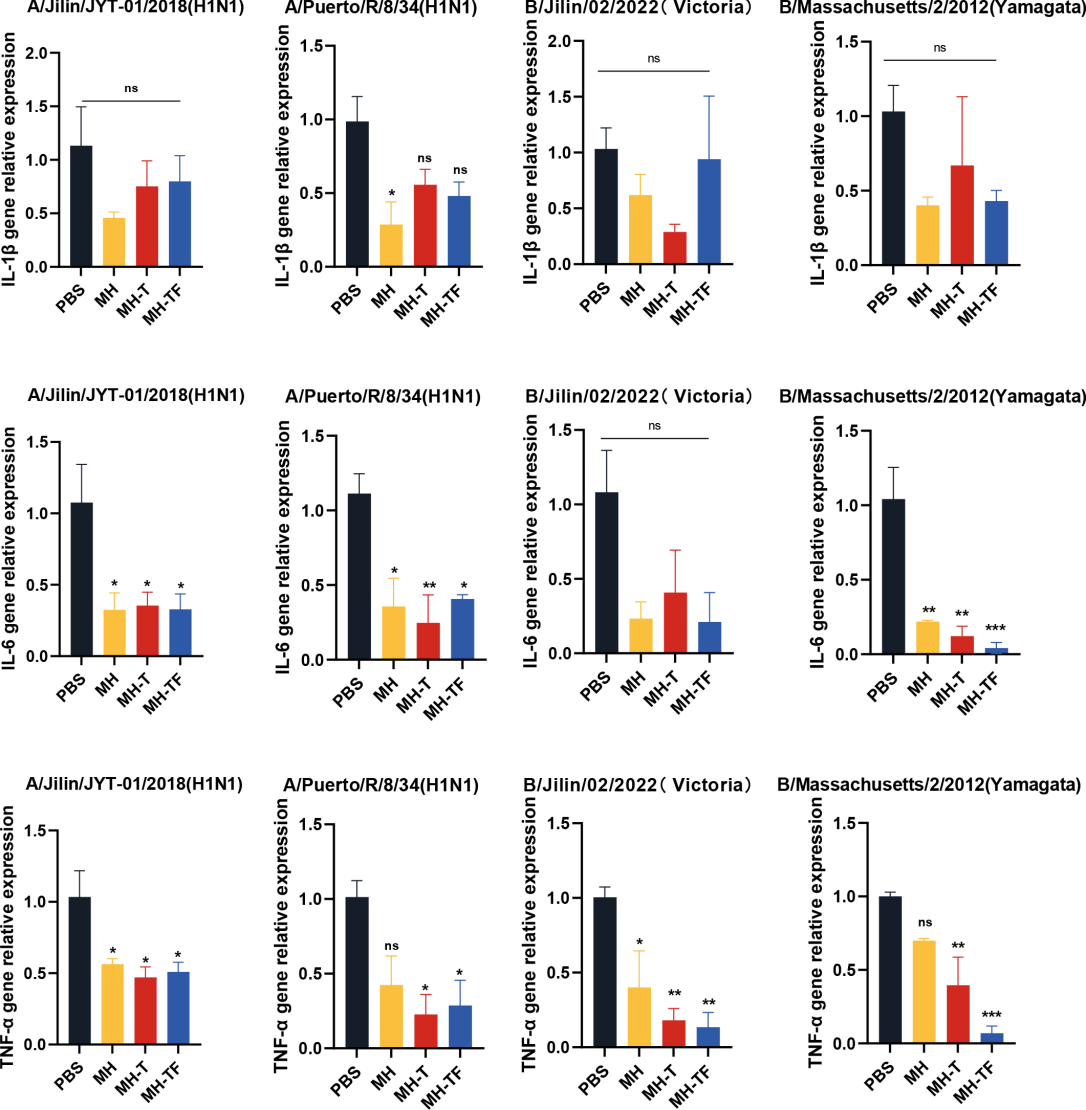
Assessment of inflammatory responses post mRNA vaccination. Transcription levels of cytokines IL-1β, IL-6, TNF-α on day 5 post-infection in lung measured using real-time qPCR. Data are mean ± SEM, analyzed using one-way ANOVA (***p < 0.001, **p < 0.01, *p < 0.05, ns *p* > 0.05).

After influenza virus infection, the expression of inflammatory cytokines can exacerbate the infection. To investigate whether vaccines can mitigate the inflammatory response, we evaluated the expression of inflammation-associated cytokines, including IL-1β, IL-6, and TNF-α (**Figure 7**). The relative real-time qPCR results revealed comparable IL-1β levels among all vaccine groups and the control group, without statistical significance. Notably, the expression of IL-6 and TNF-α genes in the vaccine group exhibited a significant reduction compared to the control group, except for IL-6 following B/Jilin/02/2022(Victoria) virus exposure.

## 4 Discussion

The major antigens on the surface of influenza viruses undergo persistent and dynamic changes, limiting the effectiveness of seasonal influenza vaccines (24, 27). The influenza vaccines targeting highly conserved HA2 and M2 epitopes have the potential to provide broad protection against antigenically transformed and drifting influenza viruses (28, 29). The epitope-based vaccine has multiple advantages, including easy recognition by the immune system, elimination of inhibitory epitopes, and stable chemical properties (30). However, single epitopes usually induce weak immune responses, so it is crucial to develop new delivery systems that can enhance the immunogenicity of conserved components (8). An ideal antigen-presenting platform is self-assembled ferritin, a common nanoparticle composed of 24 subunits (31–33). Among them, three subunits create a trimeric structure similar to the influenza virus, enhancing immunological effects by improving antigen-immune cell interaction during antigen presentation (34, 35). Ferritin is used in various vaccine platforms, especially in influenza vaccines (36, 37). Corbett et al. developed a vaccine that presents influenza HA2 trimers on ferritin nanoparticles, inducing cross-reactive antibodies in mice (25). The administration of nanoparticles containing recombinant human heavy chain ferritin and three tandem copies of M2e fusion protein resulted in robust levels of M2e-specific IgG antibodies, increased secretion of mucosal IgA antibodies, and enhanced T lymphocyte response after fatal H1N1 and H5N1 infections in nasal drop mice (38).

In this study, we developed three mRNA vaccines: MH, MH-T, and MH-TF. Immunizing mice with these vaccines induced robust humoral and cellular immunity. Cell-mediated immunity plays a crucial role in preventing influenza and providing heterologous immunity. For example, MLN-mRNA vaccines designed by Xiong et al. can effectively induce a strong T-cell response, triggering the secretion of type 1 cytokines such as IFN-γ and activating CD8^+^ T cell-mediated immune reactions (39). The protective capacity of T cells against influenza virus strains also was demonstrated in our vaccination experiments. Our results demonstrated a significant increase in spleen cells secreting the populations of CD4^+^ and CD8^+^ cells and IFN-γ in vaccinated mice after protein stimulation, indicating the vaccine’s ability to induce a robust T cell-mediated immune response.

The efficacy of our vaccine was confirmed in mouse experiments, demonstrating a significant reduction in viral load and lung tissue damage, as well as decreased levels of inflammatory cytokines. Due to the addition of the HA1 skeleton from H1 subtype influenza virus to epitopes, MH vaccine immunization can induce a relatively strong immune response and effectively protect against H1 subtype influenza virus infection. In the challenge test with influenza B virus, MH-T and MH-TF exhibited superior protective effects compared to the MH group, possibly due to the small epitope size of the influenza B vaccine which can be enhanced by multivalent form. The research suggests that the host’s inflammatory response plays a pivotal role in the pathogenesis of influenza virus infection, potentially leading to an excessive release of pro-inflammatory cytokines known as a cytokine storm or hyperacute dysregulation, ultimately resulting in fatal outcomes (40). In our study, a significant reduction in cytokine expression was observed in the MH, MH-T and MH-TF vaccine groups compared to the control group, indicating the presence of an immune protective response.

In this study, we developed a novel multi-epitope influenza vaccine aimed at achieving broad immune protection by targeting conserved viral antigens. To enhance antigen presentation efficiency and stimulate a stronger immune response, we engineered MH-T constructs containing trimer sequences and coupled them with ferritin nanoparticles to form MH-TF complexes. It is important to note that even subtle differences in the production of CD4, CD8, and IFN γ can have significant implications for protective effects against viral load and disease severity (41). In challenging experiments involving influenza B virus infection, mice vaccinated with MT-T or MH-TF vaccines exhibited more pronounced protection compared to those treated with the MH vaccine alone. Overall, optimizing antigen design and presentation can greatly enhance the efficacy of vaccines and provide a valuable strategy for developing broad-spectrum influenza vaccines.

## Data Availability

The original contributions presented in the study are included in the article/**Supplementary Material.** Further inquiries can be directed to the corresponding authors.
