# Supplementary material for: The self-assembled nanoparticle-based multi-epitope influenza mRNA vaccine elicits protective immunity against H1N1 and B influenza viruses in mice
Source: Front Immunol. 2024 Oct 8;15:1483720. doi: 10.3389/fimmu.2024.1483720 (PMC11497263; doi:10.3389/fimmu.2024.1483720)
Supplement: Supplementary file 1 [file DataSheet1.docx]

Supplementary Material

**Supplementary Table 1**. Sequences for all genes of antigens

|  | **Sequence** |
| --- | --- |
| M2e | MSLLTEVETPIRNEWGSRSNDSSD |
| HA2-H1 | DIWTYNAELLVLLENE |
| HA2-H3 | DLWSYNAELLVALENQ |
| HA2-B | DTISSQIELAVLLSNE |
| HA1-H1 | DTICIGYHANNSTDTVDTVLEKNVTVTHSVNLLEDSHNGKLCRLKGIAPLQLGKCNIAGWLLGNPECDPLLPVRSWSYIVETPNSENGICYPGDFIDYEELREQLSSVSSFERFEIFPKESSWPNHNTTKGVTAACSHAGKSSFYRNLLWLTEKEGSYPKLKNSYVNKKGKEVLVLWGIHHPSNSKDQQNIYQNENAYVSVVTSNYNRRFTPEIAERPKVRDQAGRMNYYWTLLKPGDTIIFEANGNLIAPRYAFALSRGFGSGIITSNASMHECNTKCQTPLGAINSSLPFQNIHPVTIGECPKYVRSAKLRMVTGLRNIPSIQSR |
| T4-Foldon | GSGGYIPEAPRDGQAYVRKDGEWVLLSTFL |
| Ferritin | DIIKLLNEQVNKEMNSSNLYMSMSSWCYTHSLDGAGLFLFDHAAEEYEHAKKLIIFLNENNVPVQLTSISAPEHKFEGLTQIFQKAYEHEQHISESINNIVDHAIKSKDHATFNFLQWYVAEQHEEEVLFKDILDKIELIGNENHGLYLADQYVKGIAKSRKS |

**Supplementary Table 2**. Sequences for primers used in real time RT-PCR assay

| **Primer name** | **Primer Sequence 5'-3'** |
| --- | --- |
| H1N1-JYT-F | GCATTCACCATCCACCTACT |
| H1N1-JYT-R | CCCTTCTTGATCCCTCACTTT |
| H1N1-JYT-Probe | AGATACAGCAAGAAGTTCAAGCCGGAA |
| H1N1-APR8-F | CAAGCTGGGAGGATGAACTATT |
| H1N1-APR8-R | TACTCAGTGCGAAAGCATACC |
| H1N1-APR8--Probe | ACCCGGAGACACAATAATATTTGAGGCA |
| B-Victoria-F | CTCATTTTGCAAAYCTCAAAGGA |
| B-Victoria-R | CTRTCGTGCATTATAGGAAAGC |
| B-Victoria-Probe | CTTGGGYAGACCAAAATGCACRGG |
| B-Yamagata-F | CTCATTTTGCAAAYCTCAAAGGA |
| B-Yamagata-R | CTRTCGTGCATTATAGGAAAGC |
| B-Yamagata-Probe | GGGCAGGCCAATGTGTGTGGGRACT |
| β-actin-F | AGACCTCTATGCCAACACAGT |
| β-actin-R | CATCGTACTCCTGCTTGCTGAT |
| IL-1β-F | ATCTCGCAGCAGCACATC |
| IL-1β-R | TCATCTCGGAGCCTGTAGTG |
| IL-6-F | AACAGATAAGCTGGAGTC |
| IL-6-R | TAGGTTTGCCGAGTAGAT |
| TNF-α-F | GACGTGGAACTGGCAGAAG |
| TNF-α-R | GTAGACAGAAGAGCGTGGT |
